# Submucosal technology for safe third space procedures: Amber-Red Color Imaging-guided Gastric-PerOral Endoscopic myotomy for idiopathic refractory gastroparesis

**DOI:** 10.1055/a-2673-9263

**Published:** 2025-09-04

**Authors:** Roberta Maselli, Luca Brandaleone, Roberto De Sire, Ludovico Alfarone, Davide Massimi, Cesare Hassan, Alessandro Repici

**Affiliations:** 1 Department of Biomedical Sciences, Humanitas University, Pieve Emanuele, Milan, Italy; 2 Gastroenterology, Endoscopy Unit, IRCCS Humanitas Research Hospital, Rozzano, Italy; 3 9307 Gastroenterology, IBD Unit, Department of Clinical Medicine and Surgery, Università degli Studi di Napoli Federico II, Napoli, Campania, Italy

Refractory gastroparesis is a chronic motility disorder characterized by delayed gastric emptying in the absence of mechanical obstruction. For patients unresponsive to maximal medical therapy, gastric peroral endoscopic myotomy (G-POEM) has emerged as a minimally invasive treatment targeting pyloric dysfunction 1 2 . We report the case of a 60-year-old man with idiopathic refractory gastroparesis referred for G-POEM. Radiological and motility studies confirmed delayed gastric emptying without evidence of obstruction. The procedure was performed using the Fujifilm EG-860R gastroscope (outer diameter 8.9 mm, working channel 2.8 mm) and the ELUXEO 8000 processor. HybridKnife T-type (Erbe Elektromedizin GmbH, Tübingen, Germany) was used for all procedural steps. Mucosotomy was performed along the greater curvature, followed by submucosal tunneling and full-thickness myotomy of the circular fibers of the pyloric sphincter 3 . The mucosal entry was closed using standard endoscopic clips ( Video 1 ).

Identification of tissue planes using ACI during G-POEM. Both submucosal space and vessels are emphasized to facilitate orientation during tunneling and myotomy.Video 1

Amber-Red Color Imaging (ACI), Fujifilm’s latest image-enhancement modality for third-space endoscopy, was used throughout the procedure. ACI highlights blood color contrast and improves visualization of submucosal vasculature and tissue planes, particularly the muscle, while maintaining white-light brightness 4 5 . This facilitated identification of anatomical layers and improved orientation during tunneling and myotomy ( Fig. 1 ).

**Fig. 1:**
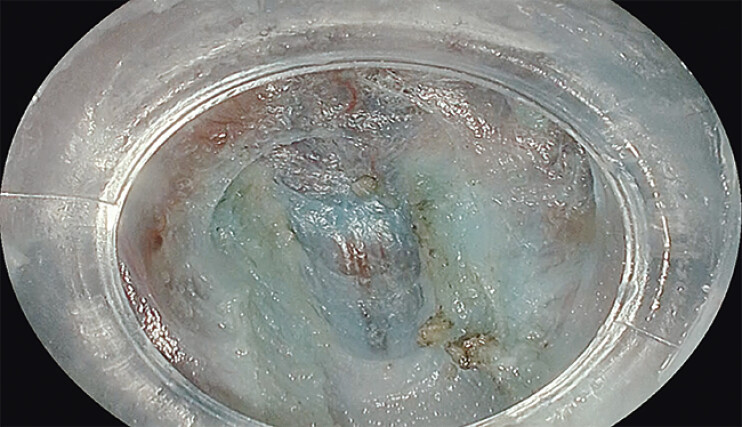
G-POEM procedure using Amber-Red Color Imaging (ACI) and a Fujifilm EG-860R gastroscope with HybridKnife T-type.

The procedure was completed uneventfully. The patient was discharged 6 hours postprocedure with no symptoms and reported clinical improvement at follow-up.

This case demonstrates the utility of ACI in enhancing visualization during G-POEM, supporting safe and efficient dissection. The integration of advanced imaging, multifunctional tools, and novel scopes may contribute to standardized and streamlined third-space endoscopy.

Endoscopy_UCTN_Code_TTT_1AQ_2AD_3AZ

## Notes

Endoscopy E-Videos https://eref.thieme.de/e-videos
*E-Videos* is an open access online section of the journal *Endoscopy* , reporting on interesting cases and new techniques in gastroenterological endoscopy. All papers include a high-quality video and are published with a Creative Commons CC-BY license. Endoscopy E-Videos qualify for HINARI discounts and waivers and eligibility is automatically checked during the submission process. We grant 100% waivers to articles whose corresponding authors are based in Group A countries and 50% waivers to those who are based in Group B countries as classified by Research4Life (see: https://www.research4life.org/access/eligibility/ ). This section has its own submission website at https://mc.manuscriptcentral.com/e-videos .
